# AGILE-ACCORD: A Randomized, Multicentre, Seamless, Adaptive Phase I/II Platform Study to Determine the Optimal Dose, Safety and Efficacy of Multiple Candidate Agents for the Treatment of COVID-19: A structured summary of a study protocol for a randomised platform trial

**DOI:** 10.1186/s13063-020-04473-1

**Published:** 2020-06-19

**Authors:** Gareth Griffiths, Richard Fitzgerald, Thomas Jaki, Andrea Corkhill, Ellice Marwood, Helen Reynolds, Louise Stanton, Sean Ewings, Susannah Condie, Emma Wrixon, Andrea Norton, Mike Radford, Sara Yeats, Jane Robertson, Rachel Darby-Dowman, Lauren Walker, Saye Khoo, on behalf of the UK NIHR community

**Affiliations:** 1 University of Southampton, Southampton, Hampshire UK; 2 Liverpool University Hospitals NHS Foundation Trust, Liverpool, UK; 3 Lancaster University and University of Cambridge, Lancaster, Lancashire UK; 4 Southampton CTU, University of Southampton, Southampton, Hampshire UK; 5 University of Liverpool, Liverpool, UK; 6 Medidata Solutions, London, UK; 7 Cancer Research UK Centre for Drug Development, London, UK

**Keywords:** COVID-19, Randomised controlled trial, Platform study, Master protocol, Phase I/II, Bayesian

## Abstract

**Objectives:**

**Phase I** - To determine the optimal dose of each candidate (or combination of candidates) entered into the platform.

**Phase II** - To determine the efficacy and safety of each candidate entered into the platform, compared to the current Standard of Care (SoC), and recommend whether it should be evaluated further in a later phase II & III platforms.

**Trial design:**

AGILE-ACCORD is a Bayesian multicentre, multi-arm, multi-dose, multi-stage open-label, adaptive, seamless phase I/II randomised platform trial to determine the optimal dose**,** activity and safety of multiple candidate agents for the treatment of COVID-19. Designed as a master protocol with each candidate being evaluated within its own sub-protocol (Candidate Specific Trial (CST) protocol), randomising between candidate and SoC with 2:1 allocation in favour of the candidate (N.B the first candidate has gone through regulatory approval and is expected to open to recruitment early summer 2020). Each dose will be assessed for safety sequentially in cohorts of 6 patients. Once a phase II dose has been identified we will assess efficacy by seamlessly expanding into a larger cohort.

**Participants:**

Patient populations can vary between CSTs, but the main eligibility criteria include adult patients (≥18 years) who have laboratory-confirmed infection with severe acute respiratory syndrome coronavirus 2 (SARS-CoV-2). We will include both severe and mild-moderate patients defined as follows: Group A (severe disease) - patients with WHO Working Group on the Clinical Characteristics of COVID-19 infection 9-point ordinal scale of Grades 4 (hospitalised, oxygen by mask or nasal prongs), 5 (hospitalised, non-invasive ventilation or high flow oxygen), 6 (hospitalised, intubation and mechanical ventilation) or 7 (hospitalised, ventilation and additional organ support); Group B (mild-moderate disease) - ambulant or hospitalised patients with peripheral capillary oxygen saturation (SpO_2_) >94% RA. If any CSTs are included in the community setting, the CST protocol will clarify whether patients with suspected SARS-CoV-2 infection are also eligible.

Participants will be recruited from England, North Ireland, Wales and Scotland.

**Intervention and comparator:**

Comparator is the current standard of care (SoC), in some CSTs plus placebo. Candidates that prevent uncontrolled cytokine release, prevention of viral replication, and other anti-viral treatment strategies are at various stages of development for inclusion into AGILE-ACCORD. Other CSTs will be added over time. There is not a set limit on the number of CSTs we can include within the AGILE-ACCORD Master protocol and we will upload each CST into this publication as each opens to recruitment.

**Main outcomes:**

**Phase I:** Dose limiting toxicities using Common Terminology Criteria for Adverse Events v5 Grade ≥3 adverse events.

**Phase II:** Agreed on a CST basis depending on mechanism of action of the candidate and patient population. But may include; time to clinical improvement of at least 2 points on the WHO 9-point category ordinal scale [measured up to 29 days from randomisation], progression of disease (oxygen saturation (SaO_2)_ <92%) or hospitalization or death, or change in time-weighted viral load [measured up to 29 days from randomisation].

**Randomisation:**

Varies with CST, but default is 2:1 allocation in favour of the candidate to maximise early safety data.

**Blinding (masking):**

For the safety phase open-label although for some CSTs may include placebo or SoC for the efficacy phase.

**Numbers to be randomised (sample size):**

Varies between CSTs. However simulations have shown that around 16 participants are necessary to determine futility or promise of a candidate at a given dose (in efficacy evaluation alone) and between 32 and 40 participants are required across the dose-finding and efficacy evaluation when capping the maximum number of participants contributing to the evaluation of a treatment at 40.

**Trial Status:**

Master protocol version number v5 07 May 2020, trial is in setup with full regulatory approval and utilises several digital technology solutions, including Medidata’s Rave EDC [electronic data capture], RTSM for randomisation and patient eConsent on iPads via Rave Patient Cloud. The recruitment dates will vary between CSTs but at the time of writing no CSTs are yet open for recruitment.

**Trial registration:**

EudraCT 2020-001860-27 14^th^ March 2020

**Full protocol:**

The full protocol is attached as an additional file, accessible from the Trials website (Additional file 1). In the interest in expediting dissemination of this material, the familiar formatting has been eliminated; this Letter serves as a summary of the key elements of the full protocol.

## Supplementary information

**Additional file 1.** Full protocol.

## Data Availability

When the database from each Candidate Specific Trial Protocol is locked, analysed and published we will make the data available to the academic community via www.clinicalstudydatarequest.com

